# Disrupting CARMA3 Signaling with Triptolide Reverses Sorafenib Resistance in Hepatocellular Carcinoma

**DOI:** 10.7150/ijms.121880

**Published:** 2025-10-20

**Authors:** Wan-Yu Wang, Tung-Wei Hsu, Yen-Hao Su, Chih-Ming Su, Tzu-Hsuan Wang, Ching-Feng Chiu, Kuei-Yen Tsai, Chih-Yang Huang, Hsin-An Chen, Po-Hsiang Liao

**Affiliations:** 1 Division of General Surgery, Department of Surgery, Shuang Ho Hospital, Taipei Medical University, New Taipei City 235, Taiwan.; 2 Graduate Institute of Medical Sciences, College of Medicine, Taipei Medical University, Taipei 110, Taiwan.; 3 TMU Research Center of Cancer Translational Medicine, Taipei Medical University, Taipei 110, Taiwan.; 4 Division of General Surgery, Department of Surgery, School of Medicine, College of Medicine, Taipei Medical University, Taipei 110, Taiwan.; 5 TMU Research Center for Digestive Medicine, Taipei Medical University, Taipei 110, Taiwan.; 6 Graduate Institute of Clinical Medicine, College of Medicine, Taipei Medical University, Taipei 110, Taiwan.; 7 Metabolic and Weight Management Center, Shuang Ho Hospital, Taipei Medical University, New Taipei City 235, Taiwan.; 8 Cardiovascular and Mitochondrial Related Disease Research Center, Hualien Tzu Chi Hospital, Buddhist Tzu Chi Medical Foundation, Hualien970, Taiwan.; 9 Department of Medical Research, China Medical University Hospital, China Medical University, Taichung 404, Taiwan.; 10 Department of Biotechnology, Asia University, Taichung 413, Taiwan.; 11 Graduate Institute of Biomedical Science, China Medical University, Taichung 404 Taiwan.; 12 Institute of Medical Sciences, Tzu Chi University, Hualien 970, Taiwan.; 13 Center for Drug Research and Development, College of Human Ecology, Chang Gung University of Science and Technology, Taoyuan 333, Taiwan.

**Keywords:** CARMA3, hepatocellular carcinoma, sorafenib, chemoresistance, triptolide

## Abstract

Hepatocellular carcinoma (HCC) is the most common primary liver malignancy and the second leading cause of cancer-related mortality. Prognostic prediction in HCC is complicated by its heterogeneity, and current treatment strategies are limited to surgical resection and targeted therapies. Sorafenib, a multi-kinase inhibitor and the first-line systemic therapy for advanced HCC, offers modest survival benefits and often induces resistance during long-term administration. Therefore, elucidating the molecular mechanisms of drug resistance is critical for improving therapeutic outcomes. Triptolide is a diterpenoid triepoxide extracted from the traditional Chinese herb Tripterygium wilfordii, exhibits potent anti-inflammatory and anti-neoplastic properties in various cancer types. CARMA3 (CARD10), a membrane-associated scaffold protein, has recently emerged as a key oncogenic regulator in solid tumors. This study demonstrates that CARMA3 contributes to chemoresistance in HCC and that triptolide enhances chemosensitivity by downregulating CARMA3 expression and promoting reactive oxygen species (ROS) accumulation. Our findings suggest that triptolide functions as a chemosensitizing agent by modulating CARMA3-mediated ROS accumulation and ferroptosis resistance, offering a novel therapeutic strategy for overcoming HCC drug resistance.

## Introduction

Hepatocellular carcinoma (HCC) is one of the most common cancers worldwide and is primarily driven by chronic inflammation, hepatotropic viral infections, or exposure to hepatotoxic agents. Primary liver cancer ranks as the fourth most frequently diagnosed malignancy and the third leading cause of cancer-related deaths globally 1-3. Despite its diverse etiologies, HCC exhibits several shared molecular characteristics, including genomic instability, dysregulated receptor tyrosine kinase (RTK) signaling, and uncontrolled cellular proliferation 4-6. Additional risk factors include alcohol abuse, non-alcoholic fatty liver disease (NAFLD), hereditary hemochromatosis, and components of the metabolic syndrome 3, 6. Although advances in diagnostic technologies and therapeutic strategies have been achieved, treatment options for HCC remain limited. Standard therapies include surgical resection, liver transplantation, and targeted therapy; however, patients with advanced-stage disease frequently exhibit resistance to conventional chemotherapeutic agents. Moreover, many anti-neoplastic drugs lack tumor selectivity and are associated with significant systemic toxicity 5. Thus, the development of more effective diagnostic biomarkers and therapeutic strategies remains an urgent clinical need.

Sorafenib, a multi-kinase inhibitor, is the first approved systemic treatment for advanced HCC. It targets the Ras/Raf/MEK/ERK signaling cascade by inhibiting Raf kinases, thereby suppressing tumor proliferation and angiogenesis. Clinical studies have demonstrated that sorafenib significantly prolongs median overall survival 7. However, resistance to sorafenib often emerges after several months of treatment, contributing to therapeutic failure and tumor progression. Understanding the molecular mechanisms underlying sorafenib resistance is essential for developing precision medicine strategies in HCC.

Triptolide, a bioactive diterpenoid triepoxide extracted from Tripterygium wilfordii Hook F (commonly known as “Thunder God Vine”), has garnered attention for its potent anti-tumor activities in various malignancies, largely mediated through induction of reactive oxygen species (ROS) accumulation and autophagy 8, 9. Triptolide exhibits nanomolar-level cytotoxicity in multiple drug-resistant cancer models and may be more cost-effective than existing chemotherapy agents 10-12. These findings suggest that triptolide could serve as a chemosensitizer, enhancing the efficacy and reducing the toxicity of existing anti-cancer drugs when used in combination. Mechanistically, triptolide differs from traditional chemotherapeutics by modulating apoptotic, oxidative, and stress response pathways. For example, triptolide has been shown to activate both intrinsic and extrinsic apoptotic pathways, including mitochondrial membrane permeabilization and caspase-3 cleavage 13, 14. Furthermore, triptolide modulates pro-apoptotic and anti-apoptotic proteins such as the Bcl-2 family and inhibitors of apoptosis (IAPs). It also induces endoplasmic reticulum (ER) stress and autophagy, which are increasingly recognized as mediators of drug resistance in cancer 15. Tumor suppressor p53 plays a dual role in DNA repair and apoptosis, and its inactivation or mutation has been associated with chemoresistance. Notably, triptolide has been reported to restore chemosensitivity through upregulation of wild-type p53 and inhibition of nuclear factor kappa B (NF-κB) signaling 16, which controls the inflammation, immunity, growth, survival, proliferation, defense against apoptosis, and anti-apoptotic proteins from Bcl-2 families in normal and tumor cells 17. However, the specific molecular mechanisms through which triptolide promotes chemosensitivity in HCC remain unclear.

CARMA3 (CARD10) is a member of the caspase recruitment domain (CARD)-containing protein family and functions as a scaffold protein that facilitates NF-κB activation through its interaction with the BCL10 and MALT1 complex, forming the CARMA-BCL10-MALT1 (CBM) signalosome 18, 19. CARMA3 is expressed in various non-hematopoietic tissues and mediates signal transduction downstream of G protein-coupled receptors (GPCRs) and RTKs. Aberrant activation of these receptors has been implicated in the pathogenesis of multiple solid tumors, including breast 20, lung, and colorectal cancers 21, 22. Additionally, CARMA3 is known to activate protein kinase C (PKC)-dependent NF-κB signaling and is responsive to phorbol esters and ionomycin stimulation. Overexpression of CARMA3 and constitutive activation of the CBM complex have been observed in several malignancies 20. The involvement of epidermal growth factor receptor (EGFR)-mediated signaling in activating CARMA3-dependent NF-κB pathways further supports its oncogenic potential 21, 22. Nevertheless, the role of CARMA3 in HCC and its relevance to triptolide-associated mechanisms remain poorly defined.

In this study, we investigated the role of CARMA3 in mediating sorafenib resistance in HCC. We demonstrate that triptolide treatment reduces CARMA3 expression, promotes ROS accumulation, suppresses antioxidant and pro-survival signaling proteins (including p-Akt, Nrf2, and HO-1), and alters the expression of key ferroptosis-associated markers, glutathione peroxidase 4 (GPX4) and ferritin heavy chain 1 (FTH1). Notably, triptolide sensitizes HCC cells to sorafenib-induced cytotoxicity, an effect that is reversed by CARMA3 overexpression. Clinically, CARMA3 is significantly upregulated in HCC tissues and strongly associated with advanced tumor stage and poor patient prognosis, as confirmed by analyses of Oncomine and TCGA datasets. Collectively, our findings establish CARMA3 as a critical regulator of sorafenib resistance and a prognostic biomarker in HCC. Importantly, triptolide exerts potent antitumor effects by disrupting CARMA3-mediated ROS accumulation and ferroptosis suppression, highlighting its potential as a chemosensitizing agent in CARMA3-targeted therapeutic strategies for HCC.

## Materials and Methods

### Cell culture

The HCC cell lines HepG2 (RRID:CVCL_0027) and Huh-7 (RRID:CVCL_0336) were obtained from ATCC. HepG2 cells were cultured with Roswell Park Memorial Institute (RPMI) 1640 medium containing 10% fetal bovine serum (FBS) and 100 IU/mL penicillin. Huh-7 cells were cultured in Dulbecco's Modified Eagle's Medium (DMEM) supplemented with 10% FBS, 100 IU/mL penicillin, and 1% non-essential amino acids.

### Whole-cell extraction

Total cellular protein was isolated using RIPA lysis buffer (Thermo Scientific, USA) supplemented with proteinase K and phosphatase inhibitors, as previously described 23, 24. Briefly, cell pellets were lysed and centrifuged at 12,000 rpm for 15 minutes at 4°C. The resulting supernatant was collected and stored at -20°C for further use.

### MTT assay

Cell viability was assessed following previous study 23. Approximately 6000 cells were planted into 96-well plates and treated with MTT buffer (0.5 mg/mL; Sigma-Aldrich, St. Louis, MO, USA). The resulting formazan crystals were dissolved in 200 μL dimethyl sulfoxide (DMSO) then measured at 570 nm by an ELISA microplate reader.

### Western blot analysis

The total protein samples concentration were identified by the Bradford assay kit (Bio-Rad, USA). Following our previous protocol 24, equal amounts of protein (20 μg) were separated by different percentage of SDS-PAGE and transferred to polyvinylidene fluoride (PVDF) membranes (Millipore, Belford, MA, USA) using the Bio-Rad system. The membranes were blocked for 1 h with blocking buffer at RT and incubated with primary antibodies (1:1000 dilution) overnight at 4°C. Following, after wash with TBST, membranes were incubated with HRP-conjugated secondary antibodies for 1 h at RT and visualized with chemiluminescent substrate (Millipore, Billerica, MA, USA). Images were captured with the GE Digital Imaging System (Commerce, CA, USA).

### Animal model

Male NU/NU nude mice (6 weeks old) were purchased from BioLASCO Taiwan (Taipei, Taiwan). All procedures were conducted in accordance with the Institutional Animal Care and Use Committee of Taipei Medical University (Approval No. LAC-2020-0394). Mice were randomly allocated into eight groups (n = 4 per group), including control and shCARMA3 controls. Each mouse received a subcutaneous injection of Huh7 cells (1 × 10^7) suspended in Matrigel and serum-free DMEM (1:1) into both hind legs. No mice were excluded from the study. Tumor tissues were partially fixed in 10% formalin for histopathology, and the remaining samples were stored at -80°C for biochemical assays. All experimental procedures, including surgery, behavioral assessments, histological evaluations, and data analysis, were conducted blinded. As this was a pilot study, no formal power calculation was performed.

### Antibodies and drugs

Primary antibodies included Akt (sc-53934), Bcl-2 (sc-7382), Bax (sc-8532), and tubulin (sc-47778), as well as phosphorylated Akt1/2/3 (sc-7985) from Santa Cruz Biotechnology (Santa Cruz, CA, USA); cleaved caspase-3 (#9664) and CARMA3 (#1656) from Cell Signaling Technology (Danvers, MA, USA); and HO-1 (ab68477), Nrf2 (ab62352), FTH1 (ab75973), and GPX4 (ab125066) from Abcam (Cambridge, UK). HRP-conjugated secondary antibodies were obtained from Santa Cruz Biotechnology. Sorafenib and other chemicals were purchased from Sigma-Aldrich (St. Louis, MO, USA).

### Reactive oxygen species (ROS) staining and detection

Cells were cultured on 8-well chamber slides (MatTek, Ashland, MA, USA) and incubated with 10 µM H₂DCFDA (Thermo Fisher Scientific, Waltham, MA, USA) for 2 h at 37°C. After washing three times with PBS, fluorescence images were captured using a Nikon fluorescence microscope (Nikon, Tokyo, Japan). ROS-positive cells were quantified from five randomly selected fields per sample using ImageJ software (NIH, Bethesda, MD, USA).

### Plasmid and shRNA transfection

A full-length human CARMA3 plasmid, kindly provided by Prof. Peter C. Lucas (E2), was subcloned into the pcDNA6 vector (Invitrogen). Two shRNA sequences targeting CARMA3 (#1: GCTCCTAGAAGTTCAGGAGAA; #2: CGCTGCTCCATGATCCTCGAT) were purchased from RNAiCore (Academia Sinica, Taiwan). Cells at ~60% confluency were refreshed with serum-containing medium 1 h before transfection. Transient transfection was carried out with plasmids or shRNAs using PureFection nanotechnology-based reagent (System Biosciences, Palo Alto, CA, USA) for 24 h, according to the manufacturer's instructions.

### Statistical analysis

All analysis experiments were performed in triplicate on three independent occasions. Statistical evaluation was conducted using one-way ANOVA followed by Student's t-test with SigmaPlot 10.0 software (Systat Software Inc., San Jose, CA, USA). Differences were regarded as statistically significant at P < 0.05, more significant at *P < 0.01, and highly significant at **P < 0.001.

## Results

### CARMA3 and triptolide modulate chemosensitivity in HCC cells

**Figure 1:**
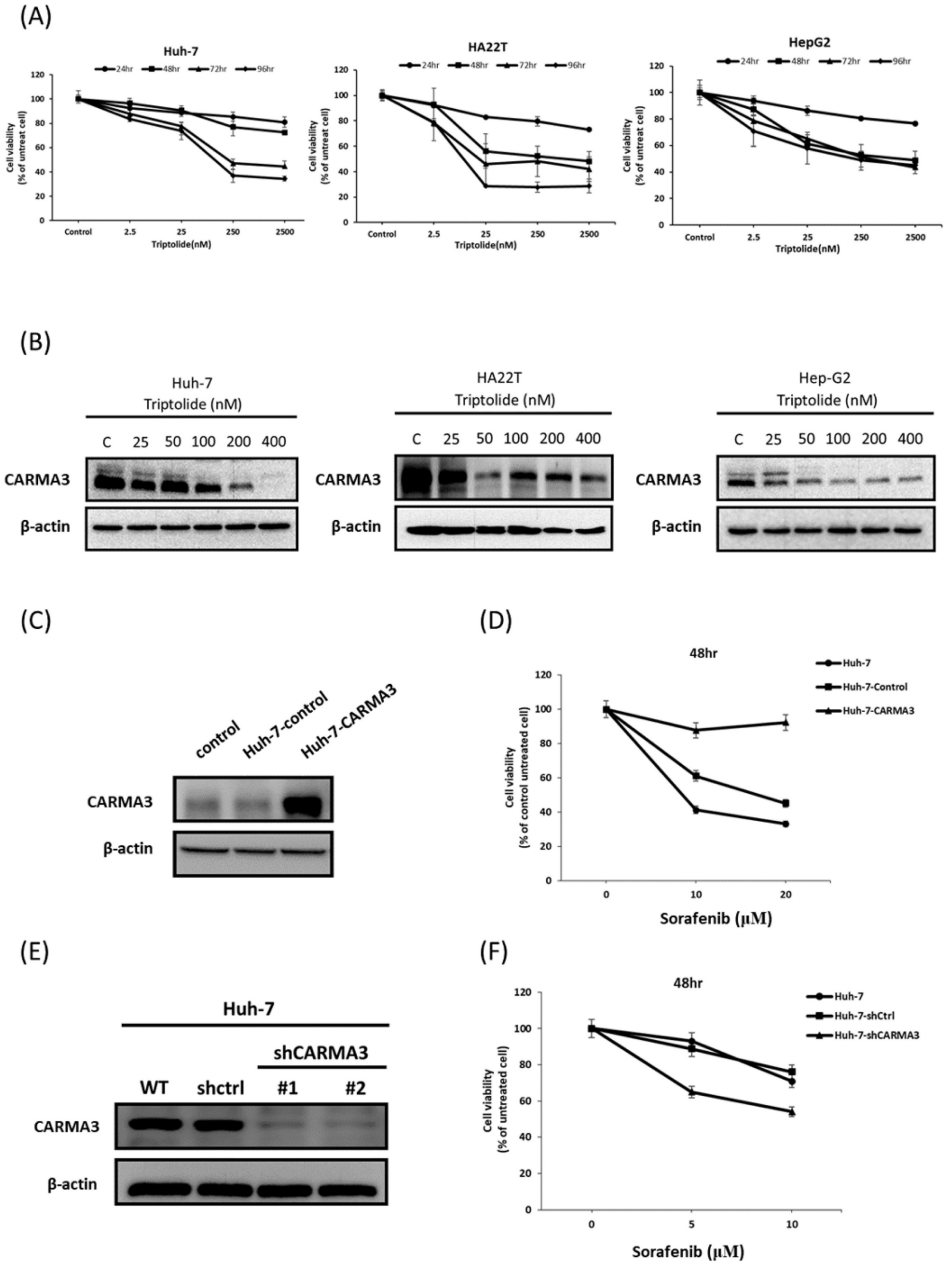
** Biological functions of triptolide and CARMA3 in hepatocellular carcinoma cells. (A)** Triptolide significantly decreased cell viability in a time- and dose-dependent manner in three HCC cell lines. **(B)** CARMA3 expression was regulated by triptolide in a dose-dependent manner, particularly in Huh-7 and Hep-G2 cells. **(C)** Overexpression of CARMA3 by plasmid was successfully established. **(D)** Overexpression of CARMA3 in Huh-7 cells significantly increased cell viability after treatment with sorafenib. **(E)** Knockdown of CARMA3 expression by shRNA was successfully achieved. **(F)** Sorafenib significantly decreased cell viability in CARMA3 knockdown cells compared with the control group.

Recent research showed triptolide had anticancer effects 10-12 and CARMA3 play an important role in liver cancer. To investigate this relationship, we treated three HCC cell lines (Huh-7, HA22T, and HepG2) with triptolide in both dose- and time-dependent manners. As shown in Figure 1A, triptolide significantly reduced cell viability across all three cell lines. We next examined whether CARMA3 contributes to triptolide-induced cytotoxicity. Triptolide treatment significantly downregulated CARMA3 protein expression in all tested cell lines, with the most pronounced and dose-dependent suppression observed in Huh-7 cells (Figure 1B). Based on this, we selected Huh-7 cells for further mechanistic studies. To clarify the role of CARMA3 in chemotherapy response, we performed overexpression and knockdown experiments. CARMA3 was successfully overexpressed via plasmid transfection (Figure 1C), while CARMA3 knockdown was achieved using two independent shRNA constructs (Figure 1D). Overexpression of CARMA3 markedly attenuated sorafenib-induced cytotoxicity (Figure 1E), whereas CARMA3 knockdown significantly sensitized cells to sorafenib (Figure 1F). These findings suggest that CARMA3 promotes sorafenib resistance in HCC cells and that triptolide may exert its anti-tumor effects, at least in part, by downregulating CARMA3 expression.

### CARMA3 functions as an oncogene in xenograft animal model

**Figure 2:**
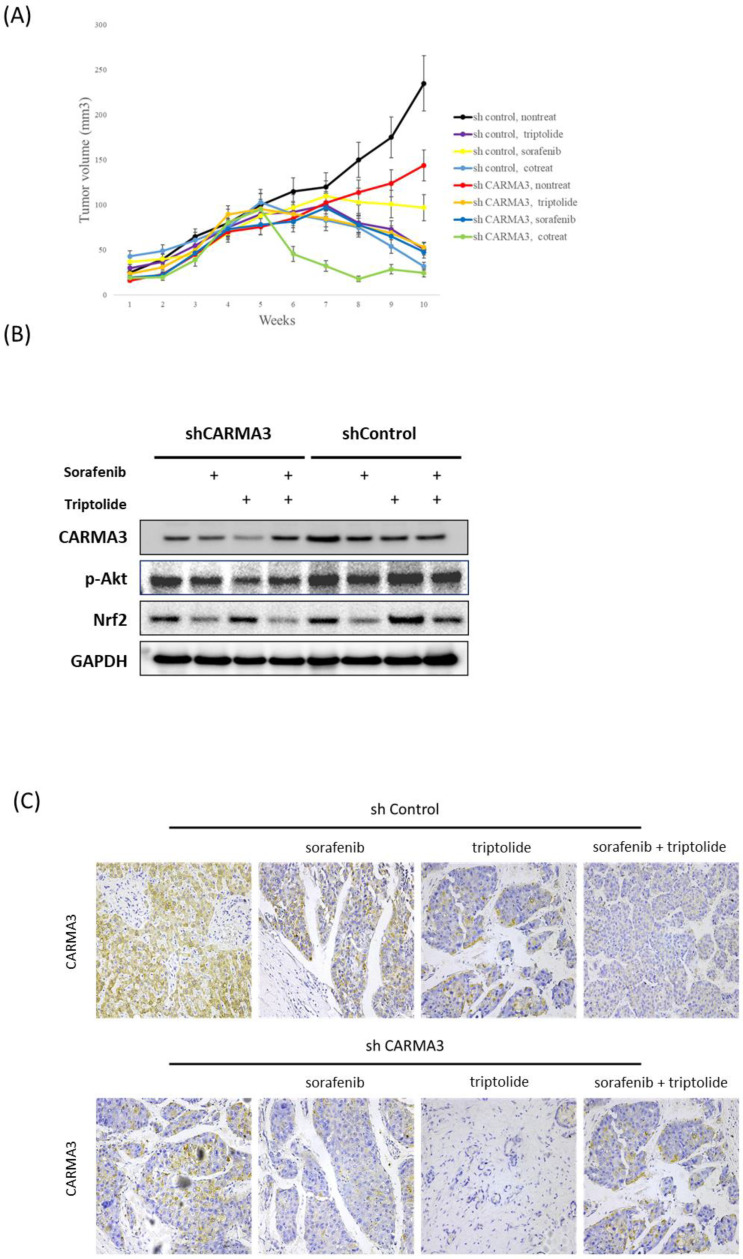
** Molecular role of CARMA3 in HCC in a xenograft animal model. (A)** Tumor volume decreased after treatment with triptolide or sorafenib in CARMA3 knockdown (shCARMA3) mice. **(B)** Triptolide significantly decreased CARMA3, phosphorylated Akt (p-Akt), and Nrf2 expression in the shCARMA3 group compared with the shControl group. **(C)** Triptolide and sorafenib regulated CARMA3 expression in tumor tissue sections, as determined by immunohistochemistry (IHC) staining.

To further investigate the oncogenic role of CARMA3 *in vivo*, we utilized a subcutaneous xenograft mouse model. Tumor volume was significantly reduced in CARMA3 knockdown (shCARMA3) mice compared with controls (Figure 2A). Notably, combined treatment with triptolide and CARMA3 knockdown yielded the most potent anti-tumor effect, outperforming either treatment alone. Western blot analysis of tumor tissues revealed that CARMA3 knockdown suppressed key survival-related proteins, including phosphorylated Akt (p-Akt) and Nrf2. Moreover, in shCARMA3 tumors, combined triptolide and sorafenib treatment further downregulated CARMA3 and p-Akt compared with controls (Figure 2B). Interestingly, triptolide exhibited stronger tumor-suppressive effects than sorafenib. Immunohistochemical staining confirmed that CARMA3 expression was markedly reduced in shCARMA3 tumors and further suppressed by triptolide, sorafenib, or combination treatment (Figure 2C). Histologically, shCARMA3 tumors displayed disorganized architecture and reduced tumor density. Collectively, these data support the role of CARMA3 as a pro-tumorigenic factor *in vivo* and demonstrate that triptolide can enhance anti-tumor efficacy by targeting CARMA3.

### Clinical relevance of CARMA3 expression in HCC by oncomine and TCGA databases

**Figure 3:**
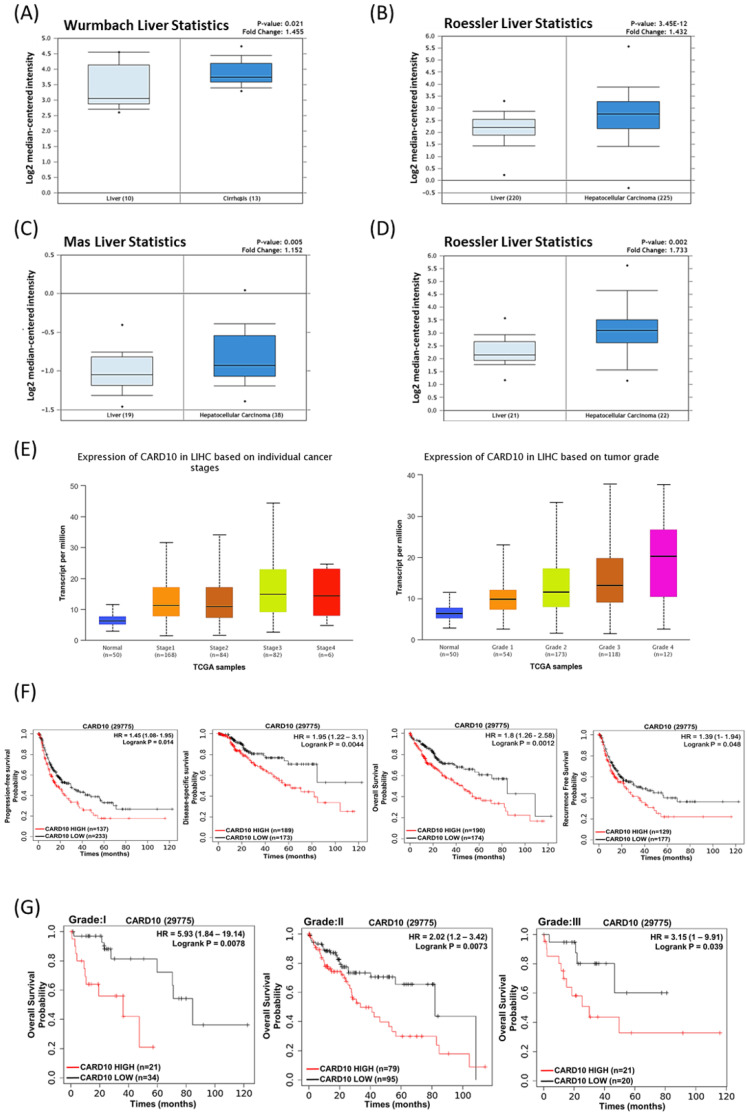
** Correlation between CARMA3 expression and liver cancer progression based on clinical analysis from Oncomine and TCGA databases. (A)** CARMA3 expression was significantly increased in liver cirrhosis compared with normal liver tissue. **(B-D)** The Oncomine database showed that CARMA3 expression in hepatocellular carcinoma was higher than in normal liver tissue. **(E)** CARMA3 expression increased with advancing liver cancer stage. **(F)** Correlation between CARMA3 expression and patient survival in liver cancer. **(G)** High CARMA3 expression was associated with lower overall survival across different liver cancer grades.

Following our previous results, that CARMA3 plays an important role in HCC cells; therefore, we want to check the role of CARMA3 in clinical database. The results showed that CARMA3 expression was up-regulated in patients with liver cirrhosis compared with normal patients (Figure 3A). Moreover, we also obtained that CARMA3 expression in HCC patients was significant increased than healthy patients (Figure 3B and Figure 3C). These results indicated that CARMA3 expression increased in HCC progression by oncomine database analysis. Following, we also check the CARMA3 (CARD10) expression in different liver cancer stages and tumor grade. After TCGA analysis we obtained that CARMA3 expression increased dependent on tumor stage (Figure 3D). Importantly, we also discovered that CARMA3 related with survival rate in HCC patients. As shown in Figure 3E, high CARMA3 expression in HCC patients lead to low progression-free survival, disease-specific survival, overall survival and recurrence free survival probability. Moreover, high expression of CARMA3 also related with low overall survival probability in different tumor grade. Taken together, clinical information indicated that CARMA3 expression will up-regulated in HCC tumor progression and is an important molecular factor related with HCC patient survival rates.

### Triptolide induces cell death by downregulating CARMA3 and promoting ROS accumulation in HCC

**Figure 4:**
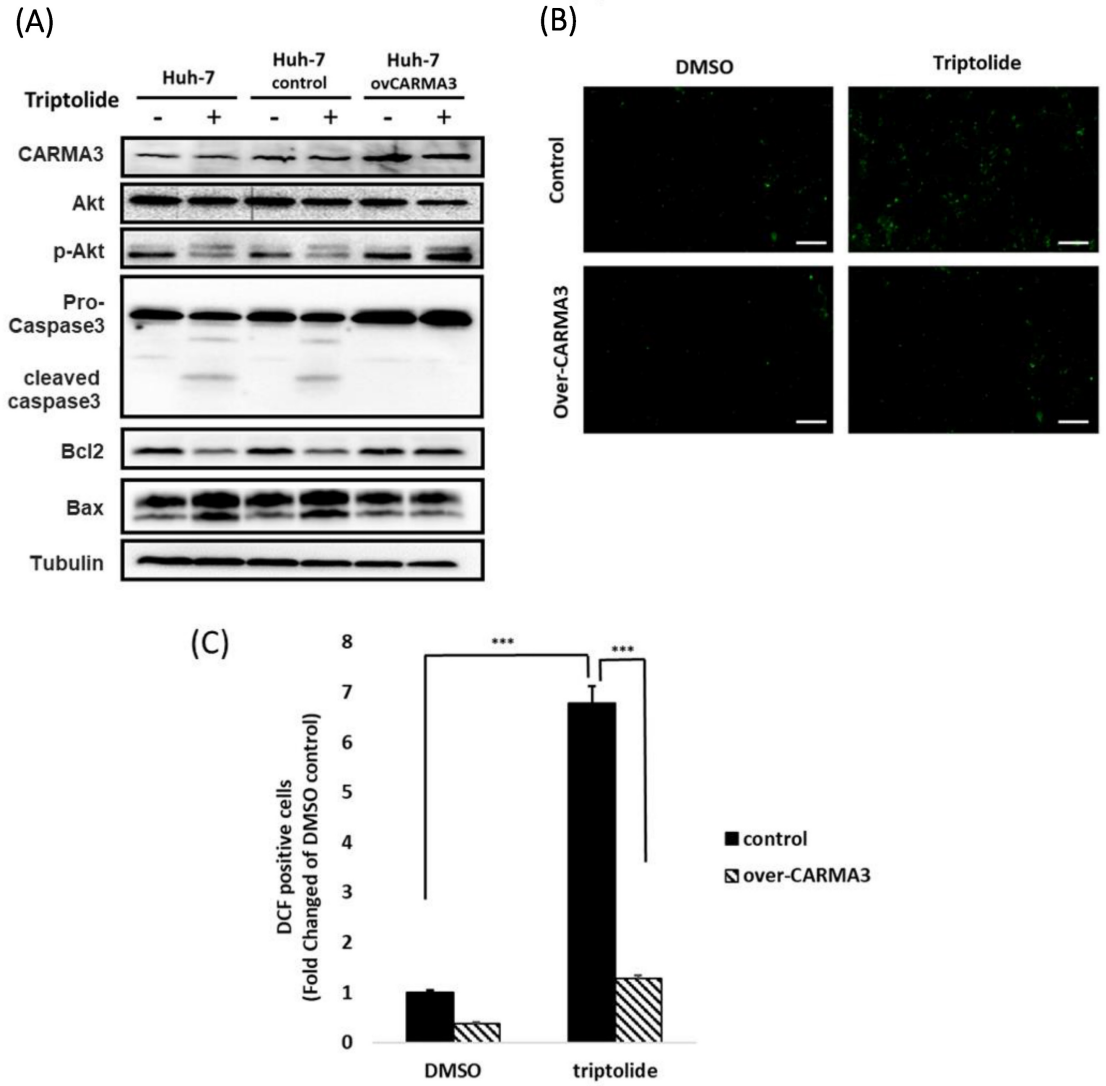
** CARMA3 reverses triptolide-induced anticancer effects in HCC cells. (A)** Triptolide induced HCC cell death by downregulating p-Akt and Bcl-2 and upregulating cleaved caspase-3 and Bax. Overexpression of CARMA3 reversed these effects, as determined by western blot analysis. **(B)** Triptolide increased intracellular reactive oxygen species (ROS) levels (green fluorescence), whereas CARMA3 overexpression attenuated ROS accumulation; scale bar: 50 μm. **(C)** Quantification of ROS levels shown in (B); ***P < 0.01.

Based on previous studies, we found that sorafenib and triptolide reduce the expression of CARMA3 in Huh-7 cells. Clinical data further indicate that CARMA3 expression correlates with HCC progression and poor prognosis. We next evaluated whether the cytotoxic effects of triptolide are mediated through CARMA3-dependent pathways. In Huh-7 cells, triptolide decreased the expression of CARMA3, p-Akt (phosphorylated Akt), and Bcl-2, while upregulating pro-apoptotic markers such as cleaved caspase-3 and Bax (Figure 4A). However, overexpression of CARMA3 attenuated these effects, suggesting a protective role for CARMA3 against triptolide-mediated apoptosis (Figure 4A). In addition, triptolide significantly increased intracellular ROS levels in a dose-dependent manner, as visualized by fluorescent staining (Figure 4B). Furthermore, ImageJ-based quantitative analysis indicated that the marked significant increase in ROS accumulation induced by triptolide in control cells was substantially attenuated by CARMA3 overexpression (Figure 4C), indicating that CARMA3 suppresses oxidative stress and contributes to chemoresistance.

### CARMA3 downregulation enhances triptolide-induced oxidative stress and ferroptosis in HCC cells

**Figure 5:**
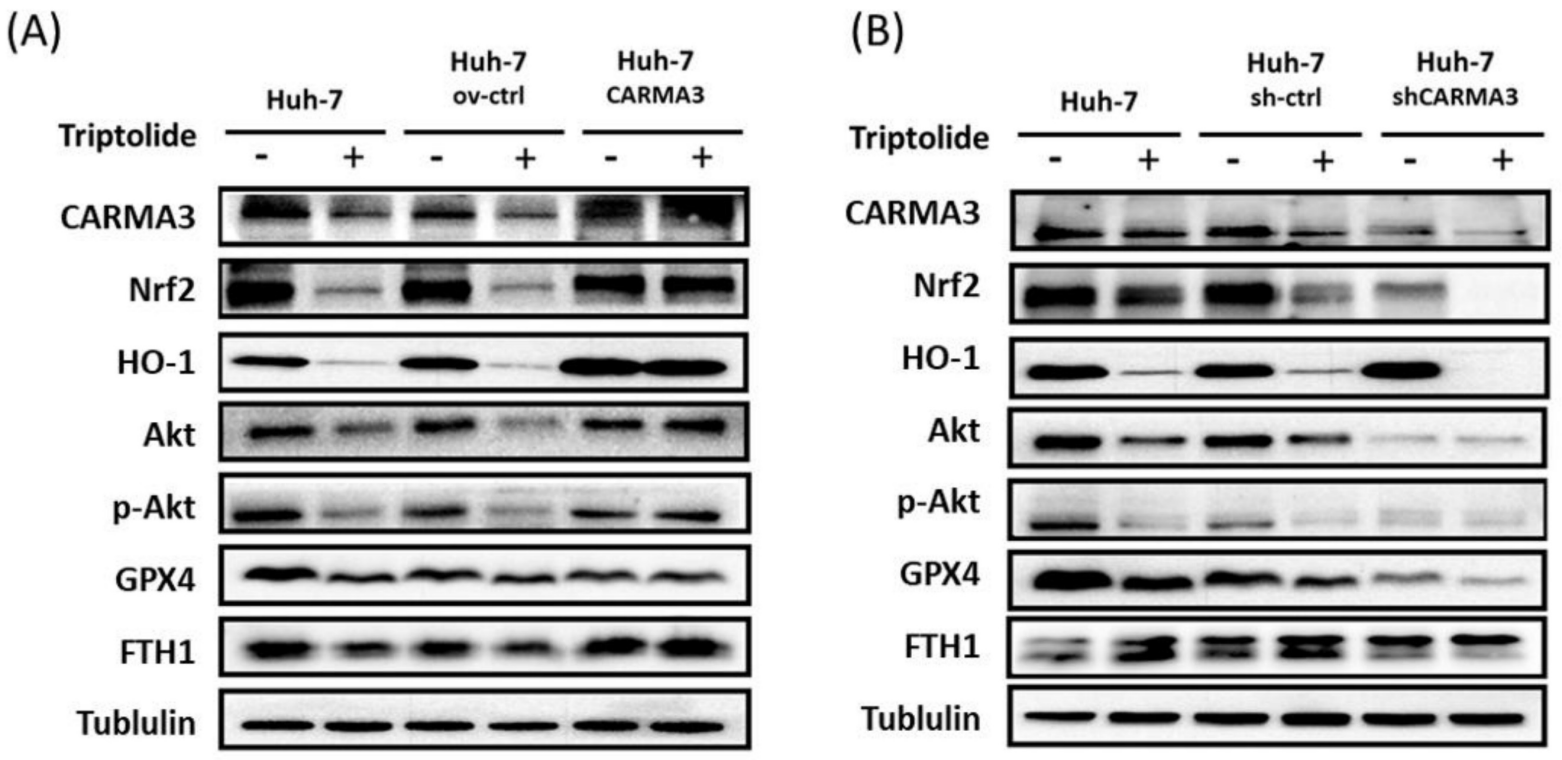
** Triptolide induces ROS accumulation and ferroptosis via CARMA3 regulation. (A)** Triptolide decreased anti-ROS proteins (Nrf2 and HO-1) and the survival protein p-Akt, while increasing ferroptosis-related proteins (GPX4 and FTH1). CARMA3 overexpression reversed these effects, as shown by western blot analysis. **(B)** Knockdown of CARMA3 attenuated the anticancer effects of triptolide.

Our previous results indicated that high expression of CARMA3 may be related to HCC cells' resistance to sorafenib, and triptolide decreased the expression of CARMA3 and induced cell injury via ROS accumulation in HCC cell lines. To further elucidate the molecular mechanisms by which CARMA3 regulates drug sensitivity, we examined the expression of redox-related and ferroptosis-associated proteins. Overexpression of CARMA3 enhanced the levels of Nrf2, HO-1, and p-Akt, and upregulated key ferroptosis suppressors GPX4 and FTH1 (Figure 5A). Conversely, CARMA3 knockdown led to a significant reduction in these proteins (Figure 5B). These data suggest that CARMA3 enhances antioxidant defenses and suppresses ferroptosis, thereby promoting cell survival and drug resistance. Triptolide-mediated downregulation of CARMA3 may restore ferroptosis sensitivity and redox imbalance, providing a novel mechanism for its anti-cancer activity.

## Discussion

The molecular functions of CARMA3 (CARD10) as a scaffold protein that integrates membrane receptor signaling with downstream activation of the NF-κB pathway. It plays a pivotal role in cancer progression, inflammation, and immune regulation 25. Moreover, a study showed that CARMA3 enhances NF-κB activity, promotes proliferation, and inhibits apoptosis in various cancers. Silencing CARMA3 has been shown to suppress NF-κB signaling, reduce tumor growth, and induce apoptotic cell death 26. These findings suggest that CARMA3 plays a pro-tumorigenic role in the progression of HCC, partly through the NF-κB signal pathway. Although these reports observed the roles of CARMA3 in oncogenesis in a variety of solid tumors and the effects of CARMA3 in HCC through the NF-κB signal pathway but whether CARMA3 involved in chemosensitivity in HCC still need more evidence to clarify. In this study, we provide new insights into the oncogenic function of CARMA3 in HCC, demonstrating its involvement in both tumor growth and chemoresistance. Our *in vitro* results obtained that CARMA3 was expressed in three HCC cell lines and we chose Huh-7, which had the highest expression of CARMA3, to hypothesize that CARMA3 might be involved in chemosensitivity. CARMA3 knockdown sensitized HCC cells to sorafenib, while its overexpression conferred sorafenib resistance. Moreover, clinical data show that high CARMA3 expression correlates with advanced TNM stage and tumor burden, and is significantly elevated in HCC tumor tissues relative to normal liver, suggesting its clinical relevance in disease progression. Previous studies have demonstrated that CARMA3 mediates the activation of G protein-coupled receptors and receptor tyrosine kinases (RTKs) in tumor pathogenesis 21 a, and its expression has also been linked to Nrf2 activation in pancreatic cancer 27. Consistent with these findings, our results indicate that modulation of CARMA3 expression influences ROS-related proteins, including Nrf2 and HO-1, as well as pro-survival proteins such as p-Akt (Figure 5). These results suggest that the regulation of CARMA3 contributes to chemoresistance in HCC through its effects on oxidative stress and survival pathways, thereby providing a mechanistic explanation for the role of CARMA3 in mediating sorafenib resistance and its reversal by triptolide treatment. Animal models are critical for clarifying the role of CARMA3 in tumor progression, and patient-derived xenograft (PDX) models are widely used to investigate the tumor microenvironment in cancer research. However, in this study we selected a xenograft animal model, which allowed us to more effectively regulate CARMA3 expression and thereby elucidate its molecular role in HCC. Using this model, we further confirmed the contribution of CARMA3 to chemoresistance *in vivo* (Figure 2). Although PDX models could provide additional insights into tumor-stromal interactions, our xenograft model offers a more practical approach for mechanistic studies. Future investigations using PDX models may help validate the clinical relevance of CARMA3 in HCC progression and therapy resistance. These findings expand upon earlier reports by suggesting that CARMA3 is not only a driver of tumorigenesis but also a key modulator of therapeutic response in HCC.

Triptolide, a diterpenoid derived from Tripterygium wilfordii Hook F, has emerged as a promising anti-cancer agent with diverse mechanisms of action, including the induction of autophagy 28, activation of NF-κB signaling pathway 29 and regulation of CaMKKβ-AMPK signaling pathway 30. Although these messages indicated that triptolide provides anti-cancer activity may through these molecular mechanisms but the role of triptolide in modulating chemosensitivity in HCC has not been fully explored. Our study reveals, for the first time, that triptolide functions as a chemosensitizer in HCC by downregulating CARMA3, thereby disrupting ROS accumulation and enhancing sorafenib-induced cytotoxicity. Mechanistically, among the three HCC cell lines (Huh-7, HepG2 and HA22T) tested, Huh-7 cells exhibited the highest CARMA3 expression. After being treated with triptolide, significant decreased CARMA3 expression hints that triptolide might be used for chemosensitizer or adjuvant therapy in chemotherapy in HCC. Subsequently, we found that triptolide decreased cell viability in Huh-7 and Huh-7 overexpressed CARMA3 (Huh-7/CARMA3) groups. However, triptolide only regulates the survival-related proteins and apoptosis-related proteins in Huh-7 control cells but induces ROS accumulation both in Huh-7 control and Huh-7/CARMA3 cells. Previous studies indicated that triptolide regulated Nrf2-driven glutathione metabolism or JNK-dependent activation of ROS accumulation leading to cell death 15. These data suggest that CARMA3 acts as a regulator of oxidative stress and that its downregulation by triptolide sensitizes cells to ROS-induced apoptosis. Although triptolide exhibited beneficial effects in overcoming chemoresistance in HCC, further evidence regarding its bioavailability and safety, as well as ethical considerations, is required before it can be advanced into clinical trials. Future studies should focus on optimizing its formulation or developing suitable drug delivery systems to improve pharmacokinetics, along with comprehensive preclinical evaluations to validate its efficacy and safety in clinically relevant models.

Ferroptosis is a regulated form of non-apoptotic cell death characterized by iron-dependent lipid peroxidation and disruption of ROS accumulation 31. Unlike apoptosis or necrosis, ferroptosis is driven by the accumulation of lethal ROS and the failure of antioxidant systems, particularly GPX4, to detoxify lipid hydroperoxides 32-34. In recent years, ferroptosis has emerged as a critical vulnerability in various malignancies, including HCC, where it plays dual roles in tumor suppression and drug resistance adaptation 35, 36. In the context of HCC, aberrant activation of antioxidant pathways such as the Nrf2-HO-1 axis, along with upregulation of ferroptosis-suppressing proteins like GPX4 and FTH1, confers resistance to oxidative stress and chemotherapy-induced cell death 35. Our findings suggest that CARMA3 upregulation increased levels of GPX4 and FTH1—two key suppressors of ferroptosis—while CARMA3 knockdown led to their downregulation. This mechanism likely contributes to the observed chemoresistance phenotype in CARMA3-overexpressing HCC cells. Importantly, we show that triptolide disrupts this protective axis by downregulating CARMA3, thereby weakening the antioxidant defense and sensitizing cells to ferroptotic stimuli. These findings are consistent with emerging literature describing ferroptosis induction as a novel therapeutic strategy in HCC, especially in cases resistant to apoptosis-inducing agents. Thus, pharmacologic downregulation of CARMA3 by triptolide may restore ferroptosis sensitivity and offer an alternative approach to overcoming sorafenib resistance.

In summary, this study identifies CARMA3 as a critical regulator of chemoresistance in HCC. We demonstrate that high CARMA3 expression is associated with poor prognosis, tumor progression, and reduced sensitivity to sorafenib. Mechanistically, CARMA3 promotes cellular survival by activating the Akt/Nrf2 antioxidant pathway and suppressing ferroptosis through upregulation of GPX4 and FTH1. Importantly, triptolide exerts potent anti-tumor activity by downregulating CARMA3 expression, enhancing ROS accumulation, and reversing chemoresistance. These effects occur both *in vitro* and *in vivo* and are particularly evident in HCC cells with elevated CARMA3 expression. Furthermore, triptolide enhances sorafenib efficacy and may function as a chemosensitizer by disrupting CARMA3-mediated redox balance and survival signaling. Our findings support CARMA3 as a potential prognostic biomarker and therapeutic target in HCC and highlight triptolide as a promising adjuvant candidate for enhancing the efficacy of current treatments, particularly in chemoresistant HCC subtypes.
